# Structural insights into hybridoma-derived neutralizing monoclonal antibodies against Omicron BA.5 and XBB.1.16 variants of SARS-CoV-2

**DOI:** 10.1128/jvi.01307-24

**Published:** 2025-01-07

**Authors:** Hengrui Hu, Chao Leng, Yanni Shu, Lu Peng, Fan Wu, Jia Liu, Xiaolu Zhang, Wei Zhou, Qinghong Xiao, Yufeng Li, Bihao Wu, Jiamei Shen, Jiang Li, Rui Gong, Bing Yan, Fei Deng, Zhihong Hu, Sheng Cao, Manli Wang

**Affiliations:** 1 State Key Laboratory of Virology, Wuhan Institute of Virology, Center for Biosafety Mega-Science, Chinese Academy of Science 74614 , Wuhan, China; 2 Hubei Provincial Center for Disease Control and Prevention 498598 , Wuhan, Hubei, China; 3 Hubei Jiangxia Laboratory, Wuhan, China; Loyola University Chicago - Health Sciences Campus, Maywood, Illinois, USA

**Keywords:** SARS-CoV-2, Omicron variants, neutralizing antibody, K18-hACE2 mouse model, cryo-EM structure

## Abstract

The emergence of novel variants of severe acute respiratory syndrome coronavirus-2 (SARS-CoV-2) continues to pose an ongoing challenge for global public health services, highlighting the urgent need for effective therapeutic interventions. Neutralizing monoclonal antibodies (mAbs) are a major therapeutic strategy for the treatment of COVID-19 and other viral diseases. In this study, we employed hybridoma technology to generate mAbs that target the BA.5 receptor-binding domain (RBD) of the SARS-CoV-2 spike protein. Through a comprehensive screening process, we identified four mAbs capable of effectively neutralizing BA.5, XBB.1.16, and related variant infections *in vitro*, among which ORB10 was found to neutralize BA.5 variants with a plaque reduction neutralization test (PRNT_50_) of 8.7 ng/mL. Additionally, competitive binding assays, sequencing of heavy and light chain variable regions, and binding kinetics characterization provided insights into the epitopes and binding affinities of the identified mAbs. Moreover, *in vivo* experiments in the K18-hACE2 mouse model demonstrated the protective efficacy of ORB10 against both BA.5 and XBB.1.16 variants. Finally, cryo-electron microscopy structural analysis of the ORB10–RBD complex identified key residues involved in the antibody–antigen interactions, providing insights into the molecular mechanisms of neutralization and immune escape of SARS-CoV-2 Omicron variants from mAbs.

**IMPORTANCE:**

The ongoing evolution of SARS-CoV-2 has led to the emergence of variants capable of evading immune responses elicited by natural infection and vaccination, especially the highly transmissible and immune-evasive Omicron variants. This study generated and characterized a panel of monoclonal antibodies (mAbs) specifically targeting the RBD of the Omicron BA.5 variant, of which the ORB10 showed efficacy against Omicron BA.5 and XBB.1.16 variants both *in vitro* and *in vivo*. Cryo-EM structural analysis further elucidated the binding epitope interactions and neutralization mechanism between ORB10 and the BA.5 RBD protein. This study enhances our understanding of antibody-mediated neutralization of SARS-CoV-2 and provides valuable insights into the development of effective therapeutic strategies to combat ongoing SARS-CoV-2 variant infections.

## INTRODUCTION

Severe acute respiratory syndrome coronavirus-2 (SARS-CoV-2), the causative agent of coronavirus disease (COVID-19), has resulted in hundreds of millions of cases since the initial COVID-19 outbreak, posing significant challenges to global public health security and the economy. Four years after its emergence, SARS-CoV-2 variants of concern (VoCs) have continued to evolve, leading to multiple infection waves worldwide. Major VoCs reported to date include Alpha (B.1.1.7) (1), Beta (B.1.351) (2), Gamma (P.1) (3), Delta (B.1.617.2) (4), and Omicron, along with its subvariants (such as BA.1, BA.2, BA.2.12.1, BA.2.75, BA.4, BA.5, BQ.1.1, XBB.1.16, XBB.1.5, EG.5, HK.3, and JN.1) (5–8). The Omicron variant, first detected in November 2021, is the most mutated VoC, with over 15 mutations occurring in its receptor-binding domain (RBD) (9). Although Omicron infections tend to cause relatively mild symptoms, this variant has a significantly enhanced ability to spread and evade the immune system (10–12).

To date, small-molecule drugs, antibodies, and vaccines against SARS-CoV-2 have been approved for marketing and have helped alleviate the symptoms of viral infection (13–21). However, due to the continual mutation of this virus, it remains challenging to completely block viral infection and transmission. Viruses evolve rapidly, with many mutations occurring at the interaction sites of monoclonal antibodies (mAbs) in the RBD, resulting in the emergence of variants capable of evading all mAbs currently available for clinical use (9, 22). Vaccines and mAbs against SARS-CoV-2 primarily target the viral spike protein, specifically the RBD region. Notably, the most potent anti-RBD mAbs bind at or near the receptor-binding motif of human ACE2 (hACE2) to block viral entry (23).

Cryo-electron microscopy (cryo-EM) and X-ray crystallography have elucidated the three-dimensional structure of the spike protein in various conformations, including the trimer before and after fusion and its binding state to the ACE2 receptor (24–27). The binding structures of different spike protein mutants to corresponding antibodies have also been elucidated, allowing antibodies to be classified into different types (28, 29). Specifically, the vast majority of mAbs targeting the RBD act by blocking the ACE2 binding motif. However, mutations such as K417N, G446S, E484A, and Q493R present in Omicron subvariants allow these strains to evade approximately 85% of these antibodies (10, 11, 25, 30, 31). The remaining mAbs, such as S304 and S309, whose epitopes do not overlap with the ACE2-binding motif, tend to exhibit broader-spectrum activity. Nevertheless, these mABs can still be evaded by Omicron subvariants harboring mutations such as G339D, N440K, and S371L (10, 11, 25, 30, 31). These structural insights have facilitated the design of immunogens for vaccine development, targeting of mAbs, identification of neutralizing epitopes, and prediction of the impact of viral mutations on immune escape from existing mAbs.

In this study, we utilized the RBD protein of SARS-CoV-2 BA.5 to immunize mice and employed hybridoma technology and enzyme-linked immunosorbent assay (ELISA) analysis to screen for mAbs that can bind to the RBD of BA.5. Overall, we identified four antibodies with high neutralizing activity against BA.5 and tested for their neutralizing activity against another seven different SARS-CoV-2 strains. Competitive binding and binding kinetics tests were then conducted. Notably, one mAb, ORB10, demonstrated the potential to protect mice from infection with BA.5 and XBB.1.16 variants. Finally, cryo-EM was used to analyze the protein structure of ORB10 binding to the BA.5 spike protein, revealing the binding site and antibody classification characteristics of ORB10.

## RESULTS

### Preliminary screening of hybridoma-derived mAbs

**Fig 1:**
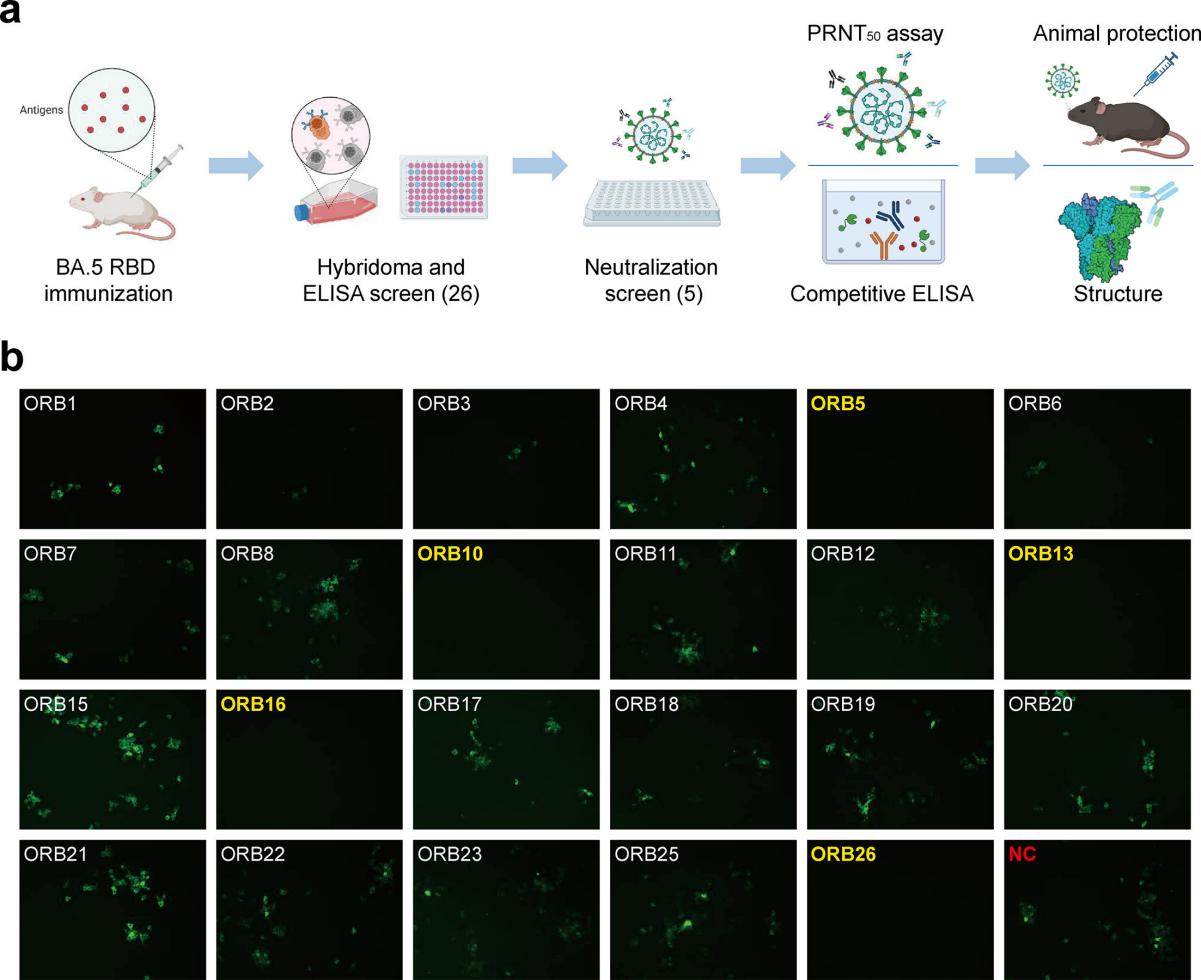
Flow charts of the study and primary screening of mAbs. (a) Flowchart illustrating the process of screening and characterizing mAbs. ELISA was used to screen cloned hybridoma cells, identifying a total of 35 mAbs. Five mAbs with high neutralization activity were further identified using microneutralization assays. The selected mAbs were subsequently subjected to a plaque reduction neutralization test (PRNT) assay, analysis of the protective effects in mice, and cryo-EM structure analysis. (b) IFA results of the primary neutralization activity screening. The mAbs that completely neutralize the BA.5 variant at a concentration of 500 ng/mL are denoted in yellow in the upper left corner; “NC” represents the negative control of the vehicle. Mouse mAbs against NP were used as the primary antibody in the IFA.

Using hybridoma technology, mAbs were generated by immunizing mice with Omicron BA.5 RBD proteins. The screening and characterization of these neutralizing mAbs are depicted in Fig. 1a. Subsequently, ELISA analysis identified 26 hybridoma cells with a strong binding affinity for the RBD. The mAbs were further purified from the hybridoma supernatants and then subjected to live virus microneutralization assay; these results revealed that five mAbs completely blocked the SARS-CoV-2 BA.5 strain at a concentration of 500 ng/mL (Fig. 1b).

### *In vitro* neutralizing activity of mAbs against different SARS-CoV-2 variants

**Fig 2:**
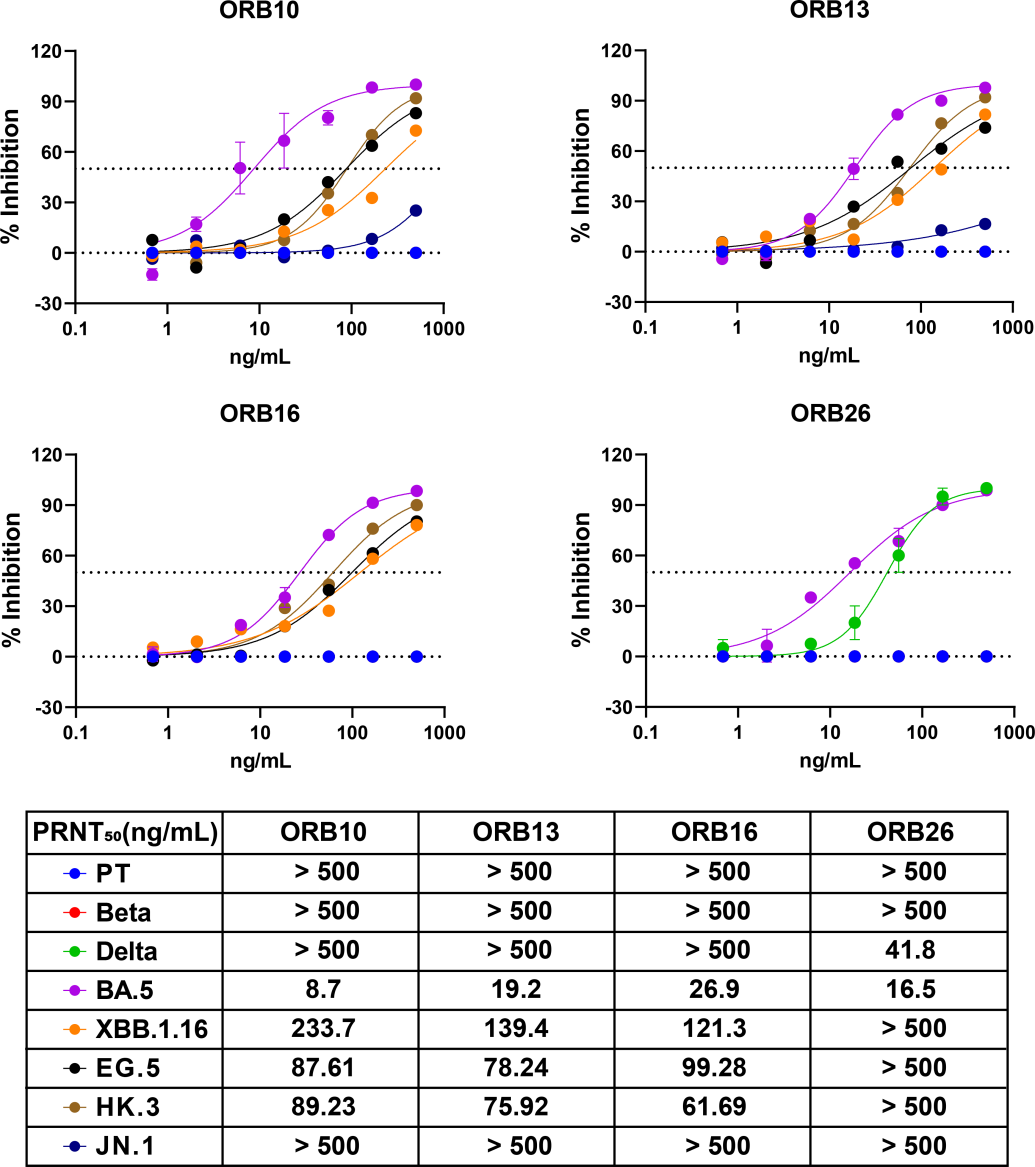
PRNT assay of ORB10, ORB13, ORB16, and ORB26 mAbs against different SARS-CoV-2 strains. Neutralizing activities of the four mAbs were detected using the PRNT assay. These mAbs were serially diluted and evaluated for neutralizing activity by counting plaque numbers. PRNT_50_ values are listed in the table below. Data are presented as the mean ± SEM of two independent experiments. PT represents the SARS-CoV-2 prototype strain.

The neutralizing activities of ORB5, ORB10, ORB13, ORB16, and ORB26 against the BA.5 strain were assessed using a plaque reduction neutralization test (PRNT) in Vero E6 cells. Notably, ORB5 exhibited the weakest neutralizing activity against BA.5, with a median plaque reduction neutralization test (PRNT_50_) value >100 ng/mL (Fig. S1); meanwhile, ORB10 demonstrated the highest neutralizing activity against the BA.5 variant, with a PRNT_50_ value of 8.7 ng/mL (Fig. 2). Further neutralization assays of ORB10, ORB13, ORB16, and ORB26 against other VoCs revealed that three antibodies—ORB10, ORB13, and ORB16—exhibited neutralizing activity against the XBB.1.16, EG.5, and HK.3 variants but not the JN.1 variant (Fig. 2) We further investigated whether these mAbs could neutralize pre-Omicron strains and found that most antibodies did not exhibit neutralizing activity against the prototype, Beta, and Delta variants of SARS-CoV-2, except for ORB26, which could neutralize the Delta variant (PRNT_50_ = 41.8 ng/mL).

### Competition assay and binding kinetics of neutralizing mAbs

**Fig 3:**
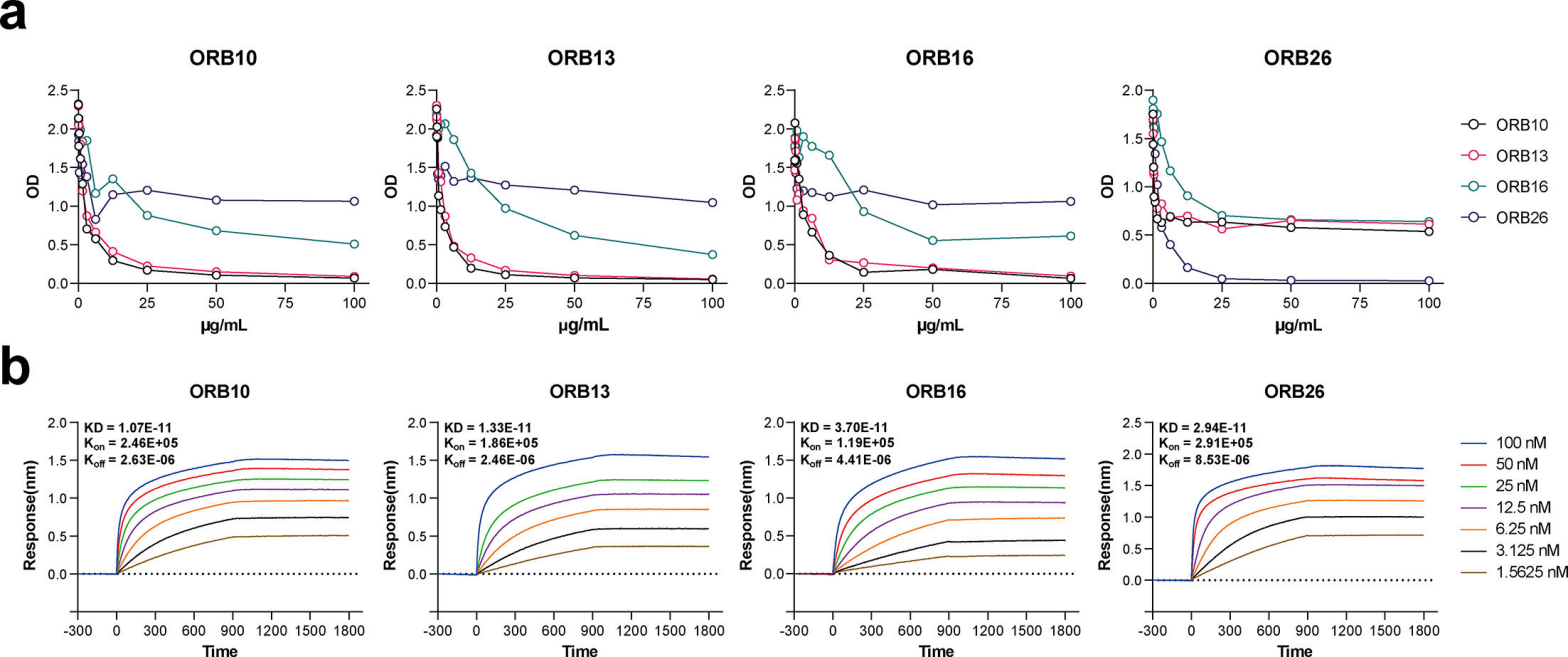
Competitive ELISA and binding kinetics of ORB10, ORB13, ORB16, and ORB26. (a) Competition between the four neutralizing mAbs. These mAbs were labeled with biotin and serially diluted, then label-free mAbs were added to compete with the labeled mAbs. (b) Binding kinetics of the four neutralizing mAbs to BA.5 RBD proteins were evaluated using BLI assays; additionally, K_on_ and K_off_ values of the mAbs were measured for each mAb.

Next, we conducted competition assays to evaluate the binding affinities of these four antibodies. A biotin-labeled mAb was added to an RBD-coated plate, followed by the addition of a label-free mAb to compete for RBD binding. Our results indicated that ORB10 and ORB13 showed strong competition against each other but only slight competition against ORB16. Meanwhile, ORB26 was only able to compete with itself (Fig. 3a). These findings suggest similar epitopes against BA.5 among the ORB10, ORB13, and ORB16, whereas ORB26 has significantly different epitopes. Further sequencing of the variable regions of these four mAbs revealed that ORB10 and ORB13 originate from the same germline and have highly similar sequences, whereas ORB16 and ORB26 were derived from distinctive germlines (Table S5 and S6). Based on the neutralization, competition, and sequencing results, the four mAbs were classified into two distinct groups, with Bin 1 being further divided into two subgroups (Fig. S3a). The binding affinities of the four mAbs to the BA.5 RBD were characterized using a biolayer interferometry (BLI) assay (Fig. 3b). Among the four mAbs, ORB10 displayed the lowest equilibrium dissociation constant (KD) value (1.07 × 10^−11^ mol/L), indicating that this mAb possessed the highest binding affinity to the BA.5 RBD. Nonetheless, all mAbs exhibited similar K_on_ and K_off_ values, suggesting high binding affinities and stable association with the BA.5 RBD.

### ORB10 protects mice against BA.5 SARS-CoV-2 infection

**Fig 4:**
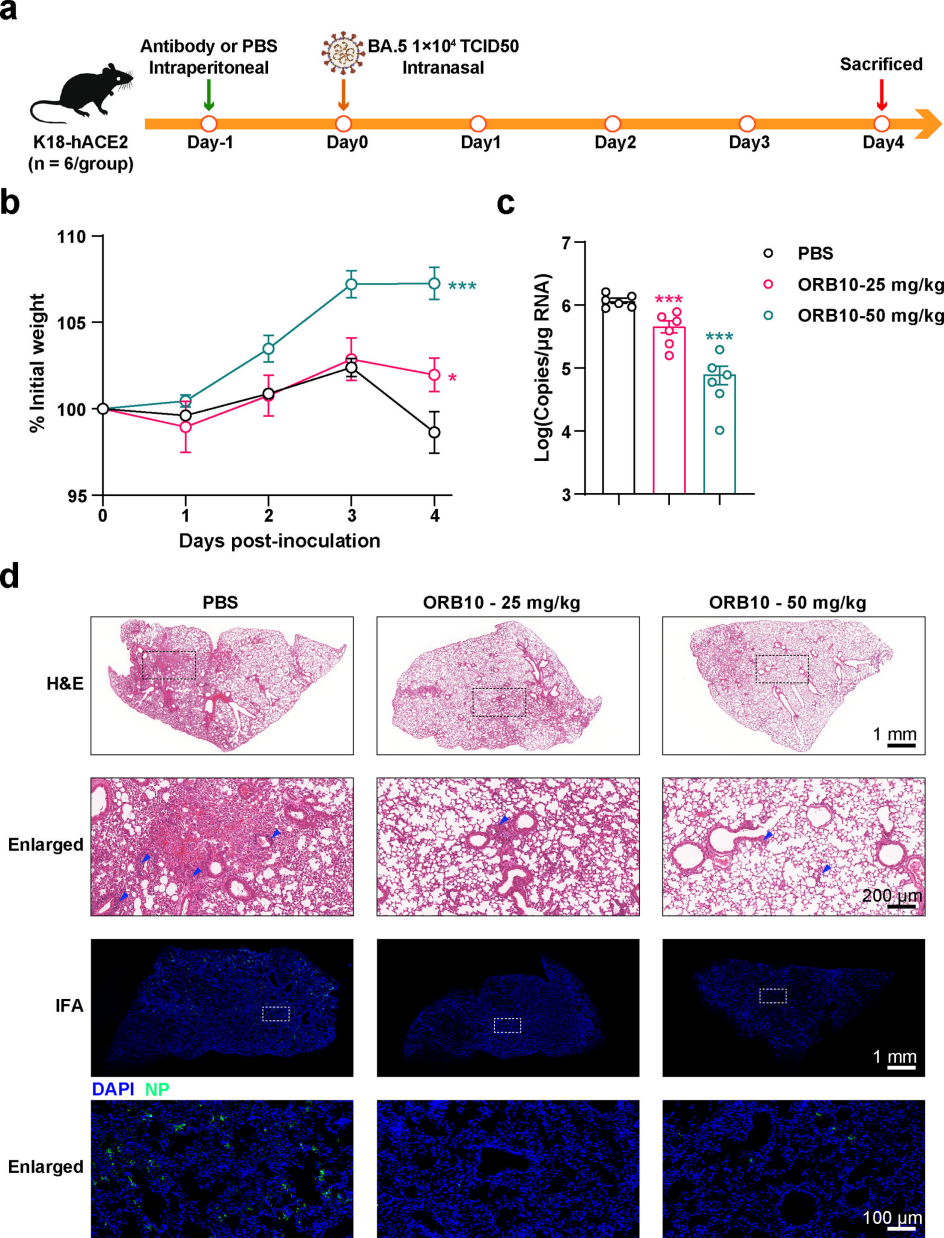
Analysis of the protective effects of ORB10 against the BA.5 variant *in vivo*. (a) Flow chart showing the analysis of the protective effects of ORB10 against BA.5 in mice. K18-hACE2 mice were intranasally infected with BA.5 at 1 × 10^4^ TCID_50_. Body weights of mice were monitored daily; mice were euthanized at 4 d.p.i. (b) Changes in the body weights of mice after infection with BA.5. Body weight is normalized to day 0 and presented as the mean ± SEM. (c) Viral copies in the lungs of mice. The same lung tissue was collected and tested for viral copy number using qRT-PCR; data are presented as the mean ± SEM. (d) Pathological analysis of the lungs of mice infected with BA.5. H&E staining and IFA were used to evaluate the pathological changes in murine lungs. Rabbit sera against SARS-CoV-2 NP protein was used as the primary antibody in the IFA. Scale bars are provided in the respective microscopic images. Dotted boxes represent the enlarged area. Blue arrows denote inflammatory cell infiltration.

*In vitro* neutralizing experiments revealed that ORB10 exhibits a high neutralizing potency against the BA.5 SARS-CoV-2 variant. Protection assays against the BA.5 variant were subsequently conducted on K18-hACE2 mice. ORB10 was injected at doses of 50 and 25 mg/kg 24 h before infection; mice were euthanized 4 days post-infection for pulmonary viral yield detection and pathological observation (Fig. 4a). Body weights of the mice in the antibody-treated groups were significantly higher than those in the PBS control group 4 days post-inoculation (*P* < 0.05), with dose-dependent effects (Fig. 4b). Consistent with these results, the viral loads in the lungs were significantly decreased in the antibody-treated groups compared with those in the PBS control group. Notably, viral loads were found to be 60.8% (*P* < 0.001) and 93.1% (*P* < 0.001) reduced in mice treated with 25 mg/kg and 50 mg/kg ORB10, respectively (Fig. 4c). Additionally, pathological analyses demonstrated that ORB10 mAbs provided significant protection against severe inflammatory cell infiltration in the lungs of mice, particularly near the bronchi and blood vessels (Fig. 4d). Meanwhile, an immunofluorescence assay (IFA) of the lung sections indicated significantly lower levels of viral nucleocapsid protein (NP) antigens in the mAb-treated mice.

### ORB10 protects mice against XBB.1.16 SARS-CoV-2 infection

**Fig 5:**
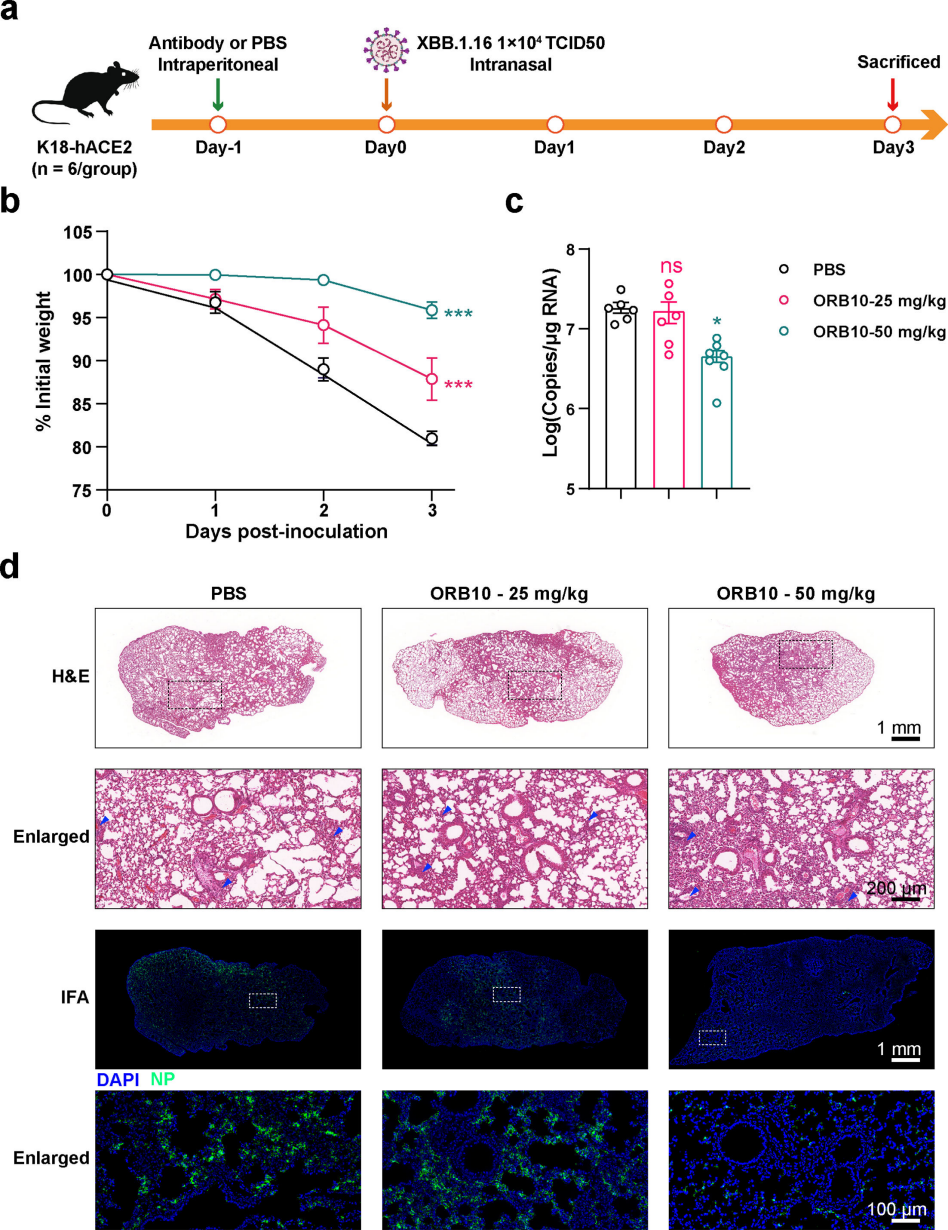
Analysis of the protective effects of ORB10 against the XBB.1.16 variant *in vivo*. (a) Flow chart showing the analysis of the protective effects of ORB10 against XBB.1.16 in mice. K18-hACE2 mice were intranasally infected with XBB.1.16 at 1 × 10^4^ TCID_50_. Body weights of mice were monitored daily; mice were euthanized at 3 d p.i. (b) Changes in the body weights of mice after infection with XBB.1.16. Body weight is normalized to day 0 and presented as the mean ± SEM. (c) Viral copies in the lungs of mice. The same lung tissue was collected and tested for viral copy number using qRT-PCR; data are presented as the mean ± SEM. (d) Pathological analysis of the lungs of mice infected with XBB.1.16. H&E staining and IFA were used to evaluate the pathological changes in murine lungs. Rabbit sera against NP was used as the primary antibody in the IFA. Scale bars are provided in the respective microscopic images. Dotted boxes denote the enlarged area. Blue arrows represent inflammatory cell infiltration.

We also evaluated the *in vivo* protective effects of ORB10 against the XBB.1.16 variant (Fig. 5a). Notably, the body weights of mice in the antibody-treated groups were significantly higher than those in the PBS control group 3 days post-inoculation, with dose-dependent effects (Fig. 5b). Moreover, viral copies in the lung tissue were found to be 24.7% (*P* > 0.05) and 90.9% (*P* < 0.05) reduced in mice treated with 25 mg/kg and 50 mg/kg ORB10, respectively (Fig. 5c). Pathological analysis revealed that high doses of ORB10 antibodies provided significant protection against severe inflammatory cell infiltration in the lungs of mice. Additionally, an IFA indicated significantly lower levels of viral NP antigens in ORB10-treated mice (Fig. 5d).

### Structural insights into ORB10 binding to the BA.5 spike trimer and interactions with the RBD

**Fig 6:**
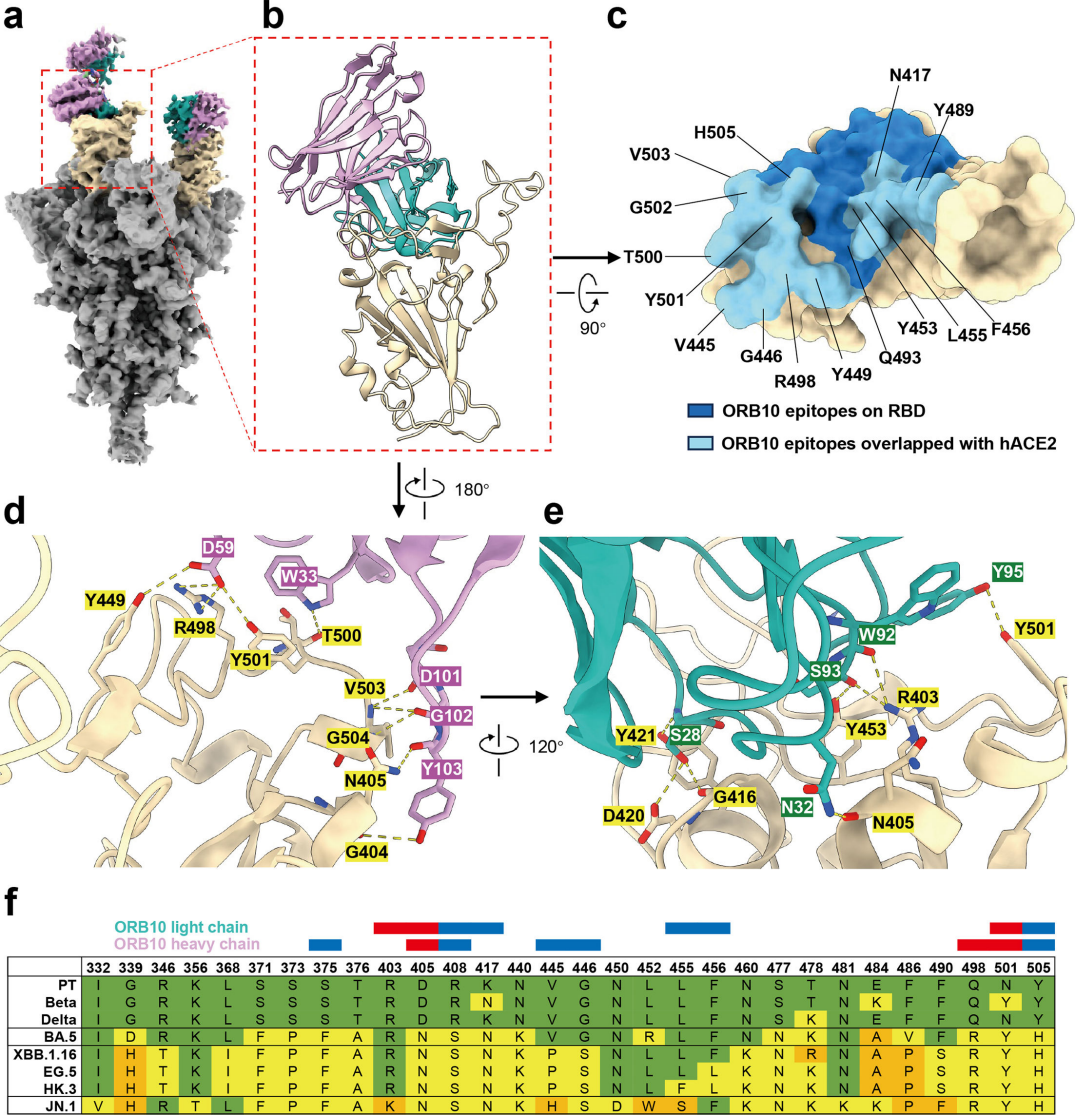
Binding stoichiometry between BA.5 S trimers and ORB10 Fab. (a) Side view of the BA.5 S trimer complexed with two ORB10 Fabs. The variable heavy and light chains of ORB10 Fab are colored purple and green, respectively; the two “up” RBDs of the BA.5 S trimer are colored in cream. (b) Magnified view of the RBD in complex with two ORB10 Fabs (PDB:8ZPP). (c) Surface illustration of the interface between the RBD and ORB10. Residues colored light and dark blue are those recognized by ORB10. Among these, residues overlapping the binding sites for ACE2 are labeled and colored light blue. (d and e) Magnified view of hydrogen bonding (yellow dashed lines) between the RBD of the BA.5 S trimer and the variable heavy chain (d) or light chain (e) of ORB10 Fab. (f) Sequence alignment of RBD mutated sites of different SARS-CoV-2 variants. The red and blue boxes above the sequence alignment represent the RBD residues involved in hydrogen bonds and interaction surface between ORB10 and BA.5 RBD, respectively.

To elucidate the molecular mechanism of ORB10-mediated neutralization, we determined the structure of the SARS-CoV-2 BA.5 spike (S) trimer in complex with the ORB10 fragment antigen-binding region (Fab) using cryo-EM. 3D reconstruction of the complex indicated that the two Fabs were bound to the RBD of the S trimer (Fig. 6a). Notably, the RBDs were in a “two up one down” conformation with two ORB10 bound to the “up” conformation but not to the “down” conformation.

Local refinement of the ORB10 Fab and RBD interface provided improved density at ~3.6 Å resolution (Fig. S2), resulting in the clear identification of the complementarity-determining regions (CDRs) of the Fab variable domains (Fig. 6b). In total, 15 residues in the RBD region at the Fab–RBD interface were determined to be involved in the interactions between the RBD of BA.5 and ACE2 (Fig. 6c), suggesting that ORB10 neutralizes the virus by blocking the binding of ACE2 receptors to the RBD. Overall, the ORB10 Fab–RBD interaction buries surface areas of 489 Å^2^ and 544 Å^2^ on the RBD *via* three CDR loops of variable heavy and light chains, respectively, primarily through hydrophobic interactions and hydrogen bonding (Table S1 and S2). In addition, 18 hydrogen bonds were identified at the ORB10 Fab–RBD interface (Fig. 6d and e; Table S3). We further aligned the mutated residues in the RBD regions from different variants and labeled the specific residues involved in the interface and hydrogen bonding between ORB10–RBD (Fig. 6f).

## DISCUSSION

During these past 4 years of SARS-CoV-2 evolution, the RBD has been a hotspot for mutations due to its role in evading the antibody response; consequently, these mutations have led to the infection of vaccinated or previously infected individuals (32). Notably, RBD mutations significantly reduce the neutralizing titers of sera from naturally infected or vaccinated individuals, resulting in reduced activity of most mAbs (11). Omicron variants are of particular concern because of their high transmissibility and ability to evade the immune response (33–35). Therefore, the development of neutralizing antibodies that can broadly protect against multiple Omicron variants and potentially other variants of SARS-CoV-2, as well as understanding the specific mechanisms of antibody–antigen interactions in this process, is crucial.

In this study, we used hybridoma technology to generate a panel of mAbs against the RBD of Omicron BA.5, which exhibited potent neutralizing activities against this strain *in vitro*. Among these mAbs, ORB10 exhibited the highest neutralizing potency, with a PRNT_50_ value of 8.7 ng/mL (Fig. 2). However, neutralization assays against other VoCs showed variable efficacy, with most mAbs showing limited or no activity against the prototype, Beta and Delta variants, except ORB26, which neutralized Delta variants. In contrast, neutralization assays against the Omicron subvariants XBB.1.16, EG.5, and HK.3 showed that these antibodies could maintain their neutralization potential against related SARS-CoV-2 variants. Meanwhile, competition assays revealed that ORB10 and ORB13 showed strong competition against themselves and ORB16, indicating overlapping or nearby epitopes (Fig. 3a). Furthermore, sequencing of mAb variable regions suggested that ORB10 and ORB13 were derived from the same germline IGHV1-80 for the heavy chain and IGKV4-53 for the light chain, whereas the ORB16 and ORB26 were derived from two distinct germlines (Fig. S3b and Table S5). In contrast, ORB26 demonstrated unique binding properties and weak competition with the other mAbs, consistent with its distinct neutralizing properties against the Delta variant. Subsequently, binding kinetic analysis demonstrated a high-affinity interaction between the identified mAbs and the BA.5 RBD, with ORB10 exhibiting the lowest KD, indicating a strong binding affinity (Fig. 3b).

*In vivo* experiments in mice demonstrated the protective effect of ORB10 against BA.5 and XBB.1.16 variants, evidenced by significantly reduced viral loads and attenuated lung pathology (Fig. 4 and 5). Furthermore, analysis of the protective effects of ORB10 in mice revealed that treatment with 25 and 50 mg/kg ORB10 reduced viral yields in BA.5-infected mice by approximately 1-fold and 10-fold, respectively. Similar reductions in viral yield were also observed in XBB.1.16-infected mice. Notably, reduced weight loss and inflammatory cell infiltration indicated that ORB10 protected the lungs from viral infection. Ultimately, this suggests that ORB10 holds potential as a therapeutic antibody against Omicron variants; nonetheless, further humanization and optimization studies are required.

Finally, structural analysis of the ORB10–RBD complex revealed the specific interaction regions between ORB10 and the viral RBD. According to the binding epitopes in the RBD, ORB10 was confirmed to be a class I antibody that binds the “up” conformation in the RBD (36). Notably, this class of mAbs encompasses several well-studied mAbs, including REGN10933, CB6, and COV2-2196 (37–39). However, the exact binding epitope of ORB10 to the RBD suggests that it binds below the center of the ACE2 binding site (Fig. 6c). This positions it within the relatively rare RBD-3 mAbs type described by Hastie et al., exhibiting a binding epitope more similar to CoVIC-080, ADI-56046, and ADG-2. However, local refinement of the binding interface of these antibodies with RBD has not yet been revealed (40, 41). Notably, we found that two antibodies, 3E2 and VacW-209, which have similar epitopes to ORB10, belong to class IV or between classes I and IV (30, 42). The differences in the overall epitopes of these antibodies were primarily attributed to variations in the binding regions of the heavy chains (Fig. S3c). Notably, the 3E2 antibody, generated from mice immunized with SARS-CoV spike proteins, exhibits cross-neutralization against both SARS-CoV and pre-Omicron variants and induces conformational changes in the RBD when combined with mainstream class I antibodies (42). The light chains of ORB10 and 3E2 both originate from the IGKV4 germline and target similar regions of the RBD, suggesting that mouse-derived IGKV4 germline genes may possess conservative binding epitope (Fig. S3c and S3d). Given that ORB10 targets the Omicron variant, although 3E2 primarily targets SARS-CoV and pre-Omicron variants, further modifications based on the sequence and structure of ORB10 and 3E2 could potentially yield antibodies with enhanced neutralizing ability and broader-spectrum activity.

ORB10 lost neutralizing activity against the prototype, Beta, and Delta variants of SARS-CoV-2. In the BA.5 strain, local refinement identified seven mutated residues (S375F, D405N, R408S, K417N, Q498R, N501Y, and Y505H) in the BA.5 strains that were involved in ORB10–RBD interactions, including three residues (N405, R498, and Y501) participating in hydrogen bonding (Fig. 6f). Four residues (V445, G446, L455, and F456) involved in the ORB10–RBD interaction, but not in hydrogen bonding, exhibited different mutation pattern in the BA.5, XBB.1.16, EG.5, and HK.3 variants. These differences could potentially explain the reduced, but not completely lost, neutralizing activity of ORB10 against XBB.1.16-related variants (Fig. 6f). Moreover, the JN.1 variant, derived from BA.2.86 with the L455S mutation, demonstrates significantly enhanced immune escape capabilities based on structural simulations (43, 44). The V445H and L455S mutation in the JN.1 variant are located at the interface of ORB10-RBD (Fig. 6f). Additionally, two residues (W92 and S93) on the light chain of ORB10 collaboratively form hydrogen bonds with R403 in the RBD, which is mutated to K403 in the JN.1 variant (Fig. 6e). These interactions may collectively lead to a significant reduction in the neutralization activity of ORB10 against JN.1. Overall, our neutralization and structural results provide insights into the important interaction sites of ORB10 with different mutant strains, offering valuable information for further antibody modifications, particularly for the subsequent JN.1-derived KP.2 and KP.3 variants.

Overall, this study reports a neutralizing antibody isolated from mice immunized with BA.5 RBD proteins that effectively binds to the RBD of the Omicron BA.5 variants. Notably, ORB10 mAbs showed efficacy against the Omicron BA.5, XBB.1.16, and related variants of both *in vitro* and *in vivo*. Cryo-EM structural analysis further elucidated the binding epitope interactions and neutralization mechanism between ORB10 and S protein. Ultimately, this study enhances our understanding of antibody-mediated neutralization of SARS-CoV-2 and provides valuable insights into the development of effective therapeutic strategies to combat ongoing SARS-CoV-2 variant infections.

## MATERIALS AND METHODS

### Cells and viruses

African green monkey kidney clone E6 cells (Vero E6; ATCC No. 1586) were cultured at 37 °C with 5% CO_2_ in Eagle’s minimum essential medium (EMEM; Gibco, Grand Island, NY, USA) supplemented with 10% fetal bovine serum (FBS, Gibco). HEK293F cells (Thermo Fisher Scientific, Waltham, MA, USA) were cultured in Freestyle 293 expression medium (Invitrogen) in an incubator at 37 °C and 8% CO_2_ with shaking at 180 rpm.

Authentic viruses of SARS-CoV-2 strains, Prototype (WIV04), Beta (B.1.351: NPRC2.062100001), Delta (B.1.617.2: IVCAS-6.7585), and Omicron (BA.5: IVCAS-6.8981, XBB.1.16: IVCAS-6.9083, EG.5: IVCAS-6.9391, HK.3: IVCAS-6.9370 and JN.1: IVCAS-6.9355), were obtained from the National Virus Resource Center (Wuhan, China). Virus amplification, determination of median tissue culture infective dose (TCID_50_/mL), and plaque assays (PFU/mL) were performed in Vero E6 cells in a biosafety level 3 laboratory at the Wuhan Institute of Virology, Chinese Academy of Sciences.

### Expression and purification of RBD proteins

The RBD proteins of the SARS-CoV-2 BA.5 strain were cloned into the pHCMV vector, then fused with a hIFNα1ss signal peptide and an S-tag at the N-terminus. HEK293F cells were transfected with polyethylenimine (PEI; Polysciences, Warrington, PA, USA); the supernatants were then collected 5 days post-transfection. S-tag beads were equilibrated with binding buffer (150 mmol/L NaCl, 20 mmol/L Tris, 0.1% Triton X-100, pH 7.5) and incubated with the supernatants containing RBD proteins at 4 °C for 2 h. The beads bound to RBD proteins were eluted with 3 mol/L MgCl_2_, followed by solution replacement with phosphate-buffered saline (PBS) using an ultrafiltration centrifugal tube (Merck, Germany). All isolated proteins were stored at −80 °C before use.

### Preparation of mAbs

Mice were immunized with the RBD protein of the BA.5 strain to prepare hybridoma cells by fusing mouse spleen cells with SP2/0 myeloma cells. After screening, 26 hybridoma cells were injected into mice to prepare ascites containing the corresponding antibodies. The caprylic acid ammonium sulfate precipitation method was then used to purify IgG antibodies. Briefly, a 4-fold volume of NaAc-HAc buffer (60 mM, pH 4.0) was added to the ascites with stirring. Subsequently, a 0.025-fold total volume of caprylic acid was added, with stirring at room temperature for 30 min. After centrifugation and filtration, a 0.35-fold volume of solid ammonium sulfate was added, and the pH value was adjusted to 7.0–8.0 using ammonia. The mixture was then centrifuged at 9,000 × *g* for 15 min at 4 C. The precipitate was resuspended in 1–2 mL PBS and dialyzed at 4°C; the PBS buffer was replaced three times at 4 h intervals. Finally, the purified antibodies were collected and stored at - 80°C.

### Sequencing of mAbs

Hybridoma cells expressing ORB10, ORB13, ORB16, and ORB26 were collected and lysed in TRIzol reagent (Invitrogen, Carlsbad, CA, USA), and the total RNA was extracted according to the manufacturer’s protocol. The sequences of mAb variable regions were amplified using the HiScript-TS 5′/3′ RACE Kit (Vazyme, China) with custom gene-specific primers for the heavy chain (CH-R: 5′-ccaggggccagtggatagacaagcttgggtgtcgtttt-3′) and light chain (CL-R: 5′-ggatacagttggtgcagcatc-3′). The amplification underwent Sanger sequencing and was subsequently analyzed using the international ImMunoGeneTics information system (https://www.imgt.org/) to confirm the germline origins.

### Neutralization analysis and immunofluorescence assay

Vero E6 cells were pre-seeded in 24-well plates (1.2 × 10^5^ cells/well). Antibodies were diluted to 1 µg/mL, mixed with equal volumes of diluted virus stock, and incubated at 37 °C for 1 h. Then, 200 µL of this mixture was added to the cells and incubated for 1 h at 37 °C to allow for virus attachment with an MOI = 0.05. Finally, the supernatants were discarded, and 500 µL of EMEM containing 2% FBS was added to the cells and further cultured for 24 h. Subsequently, the cells were fixed with 4% paraformaldehyde and permeabilized with 0.5% Triton X-100. The cells were then blocked with 5% bovine serum albumin (BSA) at room temperature for 2 h, incubated with the primary mAb against the viral nucleocapsid protein of SARS-CoV-2 (1:2,000 dilution) for 2 h, followed by incubation with the secondary antibody (Alexa 488-labeled goat anti-rabbit [1:2000; Abcam]). Images were captured using a fluorescence microscope (EVOS FL Auto, Invitrogen).

### Plaque reduction neutralization test

A PRNT was performed as previously described (45). Briefly, Vero E6 cells were pre-seeded in 24-well plates (1.2 × 10^5^ cells/well). Antibodies were 3-fold serially diluted, mixed with equal volumes of diluted virus stock, and incubated at 37 °C for 1 h. Then, 200 µL of the mixture was added to the cells and incubated for 1 h at 37 °C to enable virus attachment with a final virus titer of 100 PFU/well. Finally, the supernatants were discarded, and 500 µL of EMEM containing 2% FBS and 1% methylcellulose was added to the cells before further culturing for 2 days at 37 °C. The cells were then fixed with 4% paraformaldehyde after the medium was discarded and stained with 0.05% crystal violet. Plaque numbers were counted, and PRNT_50_ titers were calculated.

### Competitive enzyme-linked immunosorbent assay

A competitive ELISA was performed to classify the mAbs. Randomly selected mAbs were labeled with biotin (EZ-Link Sulfo NHS-LC-LC-Biotin; Thermo Fisher Scientific) and used to compete with the other mAbs. For biotin labeling, 1 µg of mAb was labeled with 120 µg of biotin (antibody–biotin) at 20–26 °C in the dark for 30 min, then dialyzed with PBS to remove residual biotin. An ELISA antigen plate was coated with 1 µg/mL RBD of BA.5 overnight in PBS at 4 °C, followed by conjugation with 100 µL of diluted antibody–biotin at 37 °C for 30 min, and incubation with 100 µL of avidin-HRP (Thermo Fisher Scientific) at 37 °C for 30 min. The OD values (OD_450_–OD_630_) were then determined; the antibody–biotin concentration that resulted in OD values ranging from 1.5 to 2.0 was used for the competition assay. Meanwhile, 50 µL of antibody–biotin and 50 µL of serially diluted label-free antibodies were mixed and added to the antigen plate. A follow-up ELISA was conducted as described above. Lower OD values indicated higher competition between the two antibodies, which can be attributed to less potent binding of the antibody-biotin complex with the RBD.

### Biolayer interferometry

The binding affinities of the mAbs to the RBD were monitored by BLI using an Octet-Red 96 device (Pall ForteBio LLC, CA,USA). Briefly, RBD was biotinylated by incubation with biotin at a molar ratio of 1:3 at 4 °C for 1 h. Residual biotin was removed by dialysis with PBS. The mAbs and RBD were then diluted in PBS containing 0.2% BSA and 0.02% Tween-20. Subsequently, biotinylated RBD proteins were loaded onto streptavidin biosensors (ForteBio, CA, USA) at a concentration of 25 µg/mL for 5 min until saturation, followed by the association and dissociation of mAbs for 10 min. These experiments were performed at 25 °C for all mAbs. Finally, the kinetics of association (K_on_) and dissociation (K_off_) were measured, and the data were processed using Octet data analysis software.

### Animal experiments

The K18-hACE2 mouse model was used to assess the protective efficacy of mAbs against SARS-CoV-2 BA.5 and XBB.1.16. Mice were intranasally infected with these SARS-CoV-2 strains; mice were intraperitoneally injected with ORB10 (25 or 50 mg/kg) as a prophylactic treatment 24 h before SARS-CoV-2 infection.

Clinical monitoring included daily assessment of body weights and observation for clinical signs indicative of infection. The lungs of mice were collected to determine viral loads and pathological changes. Viral load detection and pathological analyses were performed as previously described (45). Mouse lung tissue samples were fixed, embedded, and sectioned. The sections were then stained with hematoxylin and eosin (H&E). IFA analysis of the sections was performed using a polyclonal antibody against the SARS-CoV-2 NP as the primary antibody (1:1,000).

### Quantification and statistical analysis

Data obtained in this study were analyzed using GraphPad Prism software (v. 8.3.0). Statistical analysis included one-way ANOVA, two-way ANOVA, and nonlinear regression. Statistical significance was defined as *P* < 0.05.

### Protein production and purification

The SARS-CoV-2 S ectodomain trimer was prepared in the prefusion state as previously described (26). Briefly, a DNA fragment encoding the Omicron BA.5 S ectodomain was inserted into the pCAGGS vector containing an N-terminal signal peptide (MKWVTFISLLFLFSSAYS). A thrombin site, a T4 fibritin (foldon) domain, and a 6 × His tag were then introduced at the C-terminus. Expi293F cells were transfected with the plasmid using PEI (Polysciences) and cultured in suspension with FreeStyle 293 expression medium at 37°C in a humidified 8% CO_2_ incubator with shaking at 135 rpm. The cell culture supernatant was harvested 120 h after transfection, separated using centrifugation (10 min at 8,000 × *g* and 4°C), and filtered through a 0.45 µm filter. The S trimers were then purified by affinity chromatography using Ni-NTA resin (GenScript) and further purified by size exclusion chromatography on a Superose 6 Increase 10/300 Gl column (Cytiva).

### Production of Fab fragments from mAbs

To obtain Fab fragments, IgG was incubated with papain at a mass ratio of 1:10 in digestion buffer (2 mM EDTA, 10 mM cysteine, pH 7.5) at 37°C for 2 h. The resulting Fab fragments were purified by collecting the flow through from a protein A column; the purity of the Fab was confirmed by SDS-PAGE.

### Cryo-EM sample preparation and data collection

ORB10 Fabs were mixed with a stabilized Omicron spike protein (BA.5-Spike-6P) at a molar ratio of 3.6:1 and diluted to a final concentration of 1.0  mg/mL with PBS. After incubation on ice for 30 min, 3  µL of the mixture was loaded onto a glow-discharged holy-carbon grid (R1.2/1.3, QuantiFoil). Cryo-EM grids were prepared using an FEI Vitrobot Mark IV plunger with the following settings: chamber temperature of 16°C, chamber humidity of 100%, blotting force of 10, and blotting time of 3.5 s. Vitrified grids were loaded into a CRYO ARM 300 electron microscope (JEOL) equipped with a Gatan K3 direct electron detector. Data sets were collected using serialEM (46) at a nominal magnification of 50,000× with a defocus range of 0.5–2.5  µm in super-resolution mode, corresponding to a pixel size of 0.475  Å (Table S4). Each movie stack was dose-fractionated to 40 frames with a total electron exposure of ~40 e^-^/ Å^2^.

### Cryo-EM data processing

Frames in each movie were 2× binned, providing a pixel size of 0.95 Å, and motion-corrected using MotionCorr2 (47) in RELION (48). Following manual selection, 6,701 dose-weighted micrographs were imported into cryoSPARC (49) for contrast-transfer function estimation. Subsequently, particle selection was conducted, followed by reference-free two-dimensional (2D) classification using cryoSPARC. A total of 352,985 selected particles were subjected to *Ab-initio* reconstruction and 3D heterogeneous refinement using cryoSPARC. An additional round of homogeneous refinement was then performed, followed by local refinement of the Fab–RBD interface.

### Model building

Atomic models of ORB10 Fab and RBD were constructed based on a cryo-EM map obtained via local refinement. The cryo-EM structure of the SARS-CoV-2 RBD complexed with 14B1 Fab (PDB ID: 8I3U) was used as the starting model for subsequent model building. The initial models of Fab and RBD were rigidly fitted into the cryo-EM map using UCSF ChimeraX (50). The resulting atomic model was then manually optimized using Coot (51) and refined using phenix.real_space_refinement (52). The final model was evaluated using MolProbity (53), and hydrogen bonding networks were calculated using PDBePISA v1.52 (54).

## Data Availability

The atomic models of ORB10 Fab and RBD are available in the Protein Data Bank (PDB) under accession code 8ZPP. The sequences of mAbs are available in the supplemental material.

## SUPPLEMENTAL MATERIAL

Supplemental material jvi.01307-24-s0001.pdf Figures S1 to S3; Tables S1 to S6.

ASM does not own the copyrights to Supplemental Material that may be linked to, or accessed through, an article. The authors have granted ASM a non-exclusive, world-wide license to publish the Supplemental Material files. Please contact the corresponding author directly for reuse.
