# Supplementary material for: Structural insights into hybridoma-derived neutralizing monoclonal antibodies against Omicron BA.5 and XBB.1.16 variants of SARS-CoV-2
Source: J Virol. 2025 Jan 7;99(2):e01307-24. doi: 10.1128/jvi.01307-24 (PMC11852929; doi:10.1128/jvi.01307-24)
Supplement: Supplemental material — Figures S1 to S3; Tables S1 to S6. [file jvi.01307-24-s0001.pdf]

Supplementary Data

Structure insights of hybridoma-derived neutralizing monoclonal antibodies against BA.5 and XBB.1.16 variants of SARS-CoV-2

Hengrui Hu[a], Chao Leng[a], Yanni Shu[a], Lu Peng[a], Fan Wu[a], Jia Liu[a], Xiaolu Zhang[b], Wei Zhou[b], Qinghong Xiao[a], Yufeng Li[a], Bihao Wu[a], Jiamei Shen[a], Jiang Li[a], Rui Gong[a], Bing Yan[a], Fei Deng[a], Zhihong Hu[a], Sheng Cao[a*], Manli Wang[a, c*]

a. State Key Laboratory of Virology, Wuhan Institute of Virology, Center for Biosafety Mega-Science, Chinese Academy of Science, Wuhan, 4 30200, China
b. Hubei Provincial Center for Disease Control and Prevention, Wuhan, Hubei 430079, China
c. Hubei Jiangxia Laboratory, Wuhan, 430200, China

Hengrui Hu, Chao Leng, and Yanni Shu contributed equally to this work.

#Address correspondence to

Manli Wang, Dr. Prof.

Mailing address: Wuhan Institute of Virology, Chinese Academy of Sciences, Wuhan 430071, P.R. China. Email: wangml@wh.iov.cn; Tel/Fax: +86-27-87998086

Sheng Cao, Dr. Prof.

Mailing address: Wuhan Institute of Virology, Chinese Academy of Sciences, Wuhan 430071, P.R. China. Email: caosheng@wh.iov.cn; Tel/Fax: +86-27-87198286

Figure S1. PRNT$_{50}$ assay of ORB5 against SARS-CoV-2 BA.5 strain

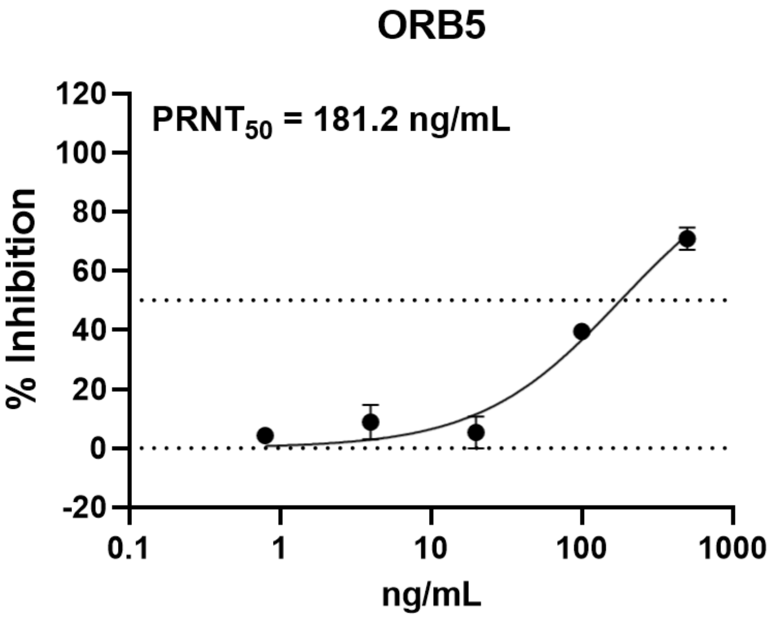

Figure S2. Cryo-EM data processing of RBD-ORB10 complex

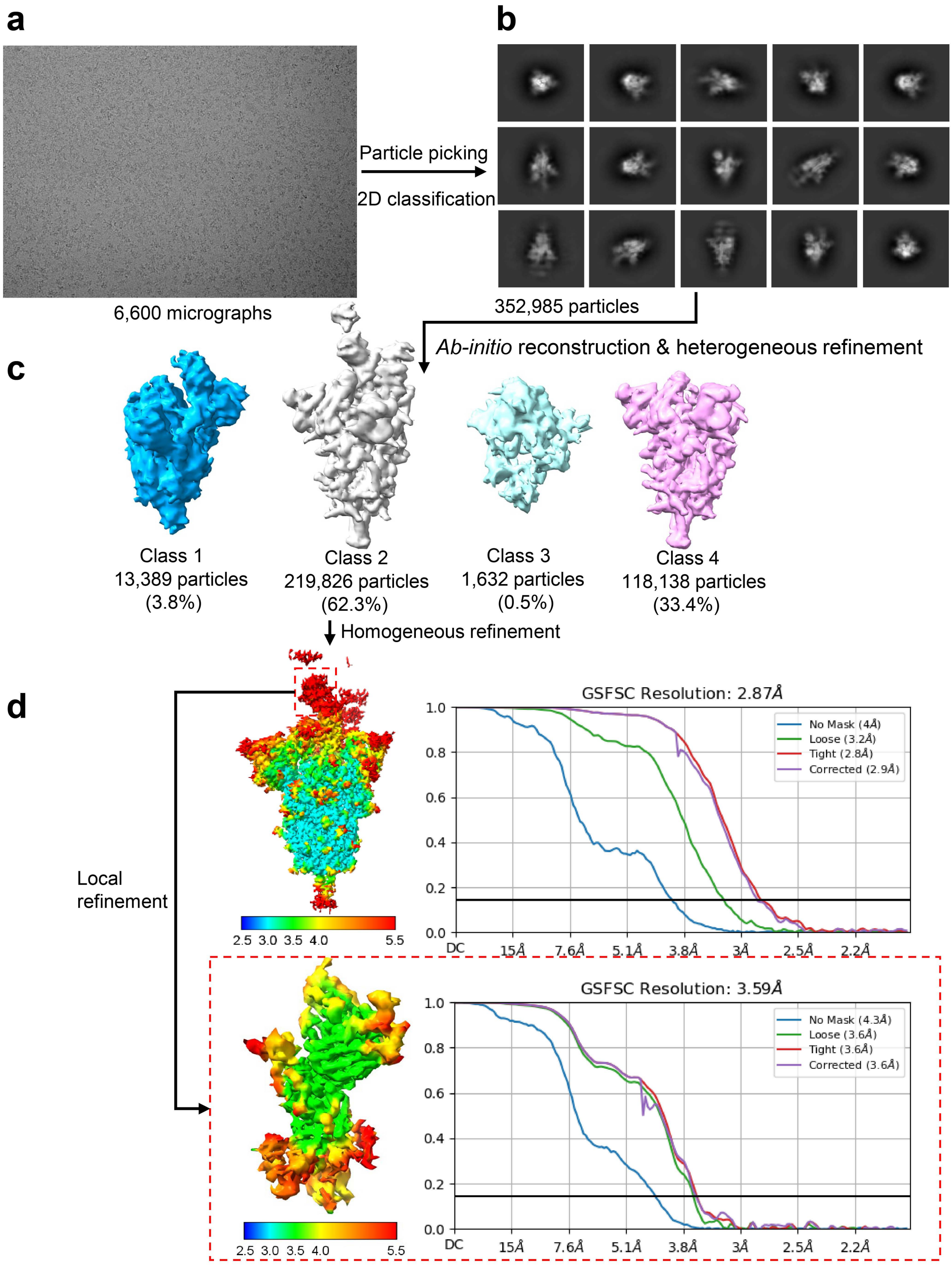

Figure S3. Binning, structure and sequence alignments of mAbs

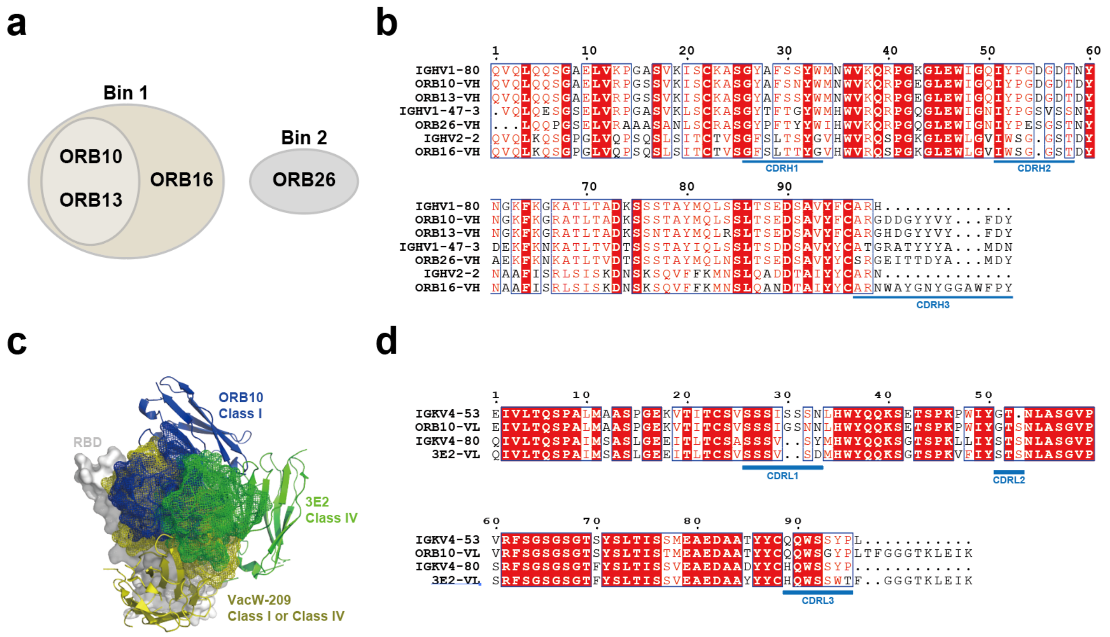

(a) Binning of four mAbs based on the neutralization, competition, and sequencing results. **(b)** Sequence alignment of the heavy chain's variable regions (VH) from ORB10, ORB13, ORB16, ORB26, and their germlines. **(c)** Structure alignment of ORB10, 3E2, and VacW-209 in complex with RBD. The light chain's variable regions (VL) of mAbs were showed with mesh. The VH were showed as cartoon. **(d)** Sequence alignment of the VL from ORB10, 3E2, and their germlines. Sequence alignments were conducted using MAFFT in MPI Bioinformatics Toolkit and visualized using ESPript3.0.

Table S1. Interactions between ORB10 heavy chain variable (VH) region and RBD

Interaction loop	Chain: Residue	Hydrogen bond	Buried Surface Area(Å²)
CDRH1			70.92
	H: Asn 31		0.37
	H: Trp 33	H	70.55
CDRH2			185.85
	H: Gln 50		15.01
	H: Tyr 52		32.40
	H: Asp 55		36.09
	H: Asp 57		45.93
	H: Thr 58		3.92
	H: Asp 59	H	52.50
CDRH3			233.04
	H: Asp 100		1.72
	H: Asp 101	H	38.00
	H: Gly 102	H	9.93
	H: Tyr 103	H	116.23
	H: Tyr 104		5.85
	H: Val 105		61.31

Table S2. Interactions between ORB10 light chain variable (VL) region and RBD

Interaction loop	Chain: Residue	Hydrogen bond	Buried Surface Area(Å²)
CDRL1			300.39
	L: Ser 26		5.83
	L: Ser 27		47.21
	L: Ser 28	H	72.74
	L: Ile 29		7.61
	L: Gly 30		37.53
	L: Ser 31		9.34
	L: Asn 32	H	65.52
	L: Asn 33		54.61
CDRL2			48.24
	L: Ser 68		19.84
	L: Gly 69		25.22
	L: Thr 70		3.18
CDRL3			195.40
	L: Trp 92	H	38.95
	L: Ser 93	H	52.00
	L: Gly 94		17.90
	L: Tyr 95	H	71.64
	L: Pro 96		14.91

Table S3. The residues/atoms involved in hydrogen bonds between Fab and RBD

RBD	Heavy chain	Light chain	Distance (Å)
Arg403(NH1)		Trp92(O)	3.38
Arg403(NH1)		Ser93(O)	2.78
Gly404(O)	Tyr103(OH)		3.76
Asn405(ND2)	Tyr103(O)		2.33
Asn405(O)		Asn32(ND2)	2.84
Gly416(O)		Ser28(OG)	2.19
Asp420(OD2)		Ser28(OG)	3.21
Tyr421(OH)		Ser28(N)	3.69
Tyr449(OH)	Asp59(OD1)		3.77
Tyr453(OH)		Ser93(O)	2.46
Arg498(NH1)	Asp59(OD2)		2.58
Arg498(NH2)	Asp59(OD1)		2.69
Thr500(O)	Trp33(NE1)		2.85
Tyr501(OH)	Asp59(OD2)	Tyr95(OH)	3.09/2.99
Val503(N)	Asp101(O)		3.06
Val503(N)	Gly102(O)		3.68
Gly504(N)	Gly102(O)		3.30

Table S4. Cryo-EM data collection, refinement, and model validation statistics

	**RBD-ORB10 Complex PDB: 8ZPP**
**Data collection and processing**	
Microscope	JEOL CRYO ARM 300
Magnification	50,000 ×
Voltage (kV)	300
Frames per stack	40
Total dose per movie ($e^-/Å^2$)	40
Defocus range (μm)	-0.5 to -2.5
Pixel size (Å)	0.475
Symmetry imposed	C1
Final particle images (no.)	219,826
Map resolution (Å)	3.6
FSC threshold	0.143
**Model Refinement**	
Model resolution (Å)	3.6
FSC threshold	0.143
Map sharpening B factor ($Å^2$)	-147.2
Model composition	
Non-hydrogen atoms	3,271
Protein residues	420
Ligand	0
B factors($Å^2$) (mean)	
Protein	63.94
Ligand	0
R.m.s. deviations	
Bond length (Å) (# > 4σ)	0.008 (0)
Bond angles (°) (# > 4σ)	0.749 (0)
**Validation**	
MolProbity score	1.88
Clash score	12.24
Poor rotamers (%)	0.00
Ramachandran plot	
Favored (%)	95.89
Allowed	4.11
Disallowed	0.00

Table S5. The germline origin and CDR3 sequence of four mAbs

mAb	Heavy V Gene	Heavy J Gene	CDRH3	Light V Gene	Light J Gene	CDRL3
**ORB10**	IGHV1-80	IGHJ2	ARGDDGYYVYFDY	IGKV4-53	IGKJ2	QQWSGYPLT
**ORB13**	IGHV1-80	IGHJ2	ARGHDGYYVYFDY	IGKV4-53	IGKJ2	QQWSGYPLT
**ORB16**	IGHV2-2	IGHJ3	ARNWAYGNYGGAWFPY	IGKV1-99	IGKJ5	FQSNYLPLT
**ORB26**	IGHV1-47-3	IGHJ4	WGQGTSVTVSS	IGKV12-98	IGKJ1	QQLSSPPLT

Table S6. Heavy chain and light chain variable region sequence of mAbs

mAb region	FR1~FR4 amino acid sequence
**ORB10-VH**	QVQLQQSGAELVRPGSSVKISCKASGYAFSNYWMNWVKQRPGEGLEWIGQIYPGD GDTDYNGKFKGRATLTADKSSSTAYMQLSSLTSEDSAVYFCARGDDGYYVYFDYW GQGTTLTVSS
**ORB10-VL**	EIVLTQSPALMAASPGEKVTITCSVSSSIGSNNLHWYQQKSETSPKPWIYGTSNLASG VPVRFSGSGSGTSYSLTISTMEAEDAATYYCQQWSGYPLTFGGGTKLEIK
**ORB13-VH**	QVQLQQSGAELVRPGSSVKISCKASGYAFSSYWMNWVKQRPGEGLEWIGQIYPGD GDTDYNGKFKGRATLTADKSSNTAYMQLRSLTSEDSAVYFCARGHDGYYVYFDYW GQGTTLTVSS
**ORB13- VL**	EIVLTQSPALMAASPGEKVTITCSVSSSIGSNNLHWYQQKSDTSPKPWIYGTSNLASG VPVRFSGSGSGTSYSLTISTMEAEDAATYYCQQWSGYPLTFGGGTKLEIK
**ORB16-VH**	QVQLKQSGPGLVQPSQSLSITCTVSGFSLTTYGVHWVRQSPGKGLEWLGVIWSGGS TDYNAAFISRLSISKDNSKSQVFFKMNSLQANDTAIYYCARNWAYGNYGGAWFPY WGQGTLVTVSA
**ORB16- VL**	DVVLTQTPLSLPVNIGDQASISCKSTKSLLNSDGFTYLDWYLQKAGQSPQLLIYLVS NRFSGVPDRFSGSGSGIDFTLKISRVEAEDLGVYYCFQSNYLPLTFGAGTKLELK
**ORB26-VH**	LQQPGSELVRAAASANLSCRASGYPFTYYWIHWVKQRPGQGLEWIGNIYPESGSTN YAEKFKNKATLTVDTSSSTAYMQLNSLTSEDSAVYYCSRGEITTDYAMDYWGQGTS VTVSS
**ORB26- VL**	DIQMTQSPASQSASLGESVTITCLASQTIGTWLAWYQQKPGKSPQLLIYAATSLADG VPSRFSGSGSGTKFSFKISSLQAEDFVTYYCQQLSSPPLTFGGGTKLEIK
